# Myasthenia Gravis Masquerading as Status Asthmaticus

**DOI:** 10.1155/2021/6959701

**Published:** 2021-12-28

**Authors:** Neha Pirwani, Shayna Wrublik, Shashikanth Ambati

**Affiliations:** Affiliated with Albany Medical Center, Albany, NY, USA

## Abstract

Myasthenia gravis, an autoimmune disorder of neuromuscular transmission, can lead to varying degrees of weakness and fatigability of the skeletal musculature. Juvenile myasthenia gravis accounts for 10–15% of all cases of myasthenia gravis. The clinical presentation of juvenile myasthenia gravis varies tremendously, which presents itself as a diagnostic challenge for clinicians. We report a case of a 15-year-old female with mild intermittent asthma presenting with shortness of breath. Acute onset of dyspnea is a common chief complaint amongst the pediatric population with a broad differential diagnosis. Our patient was presumptively treated for status asthmaticus and required invasive mechanical ventilation. After extubating, the patient showed persistent ptosis, which led to the eventual work-up of myasthenia gravis. Upon further review, this patient had months of intermittent symptoms including ptosis and fatigue which went previously undiagnosed. This case demonstrates that dyspnea in an asthmatic can occur from nonairway processes and, if missed, may result in overtreatment of asthma or delayed diagnosis of an important neuromuscular process.

## 1. Introduction

Juvenile myasthenia gravis (MG), a disorder of neuromuscular transmission, occurs in the pediatric population before the age of 19 years and causes dysfunction of acetylcholine receptors (AChR), leading to varying degrees of fluctuating weakness of affected muscles [1]. Children present with a variety of symptoms including limb or facial weakness, difficulty swallowing, ptosis or strabismus, and respiratory failure, further complicating diagnostic work-up and treatment of MG. Children and families seek treatment from various pediatric specialists with nonspecific and, often, changing symptomology. Late diagnosis contributes to less optimal care and lower remission rates [2]. To achieve earlier detection, pediatric providers should have a good understanding of various symptoms for which patients are treated and the pattern of missed diagnosis and/or delayed diagnosis for such cases.

We report a case of undiagnosed myasthenia which came to medical attention by myasthenic crisis precipitated by treatment of asthma in an adolescent female. This case demonstrates that dyspnea in an asthmatic can occur from nonairway processes and, if missed, may result in overtreatment of asthma or delayed diagnosis of an important neuromuscular process.

## 2. Case Presentation

An adolescent female with a past medical history of mild intermittent asthma presented to the emergency room (ER) with acute respiratory failure concerning for asthma exacerbation. Her initial symptoms included five days of worsening shortness of breath, dry cough, mild rhinorrhea, back pain, and intermittent diffuse headaches. She had no previous hospitalizations for asthma and uses albuterol as needed; however, no albuterol treatments were given at home as there was no appreciable wheezing. She does not require controller medication. She denied cigarette smoking, vaping, sick contacts, or COVID-19 exposures. In the ER, her pediatric assessment appreciated inspiratory and expiratory wheezes, diminished breath sounds, and subcostal retractions. She received 2 hours of continuous albuterol-ipratropium, magnesium sulfate 2 grams, and dexamethasone 16 milligrams for suspected diagnosis of status asthmaticus. She was started on continuous positive airway pressure (BiPAP) with 6 cmH_2_O. Her respiratory support escalated to bilevel positive airway pressure of 12/5 cmH_2_O. Initial labs demonstrated an arterial pH equal to 7.31, arterial pCO_2_ equal to 79 mmHg, and arterial bicarbonate equal to 39 mmol/L, concerning for primary respiratory acidosis with uncompensated metabolic alkalosis. The basic metabolic panel showed an elevated serum bicarbonate (34), indicating chronic renal compensation The rest of her labs were unremarkable, including a chest X-ray showing no focal opacities, effusions, or pneumothorax. Apart from nebulization with bronchodilators, magnesium, and steroids, the patient did not receive any antibiotics during this admission.

She was admitted to the Pediatric Intensive Care Unit (PICU) where her respiratory status declined despite receiving interventions of continuous albuterol, dexamethasone, magnesium sulfate, terbutaline, and ketamine. While in the PICU, her exam revealed limited air entry in the lungs bilaterally. Her respiratory support escalated from BiPAP to invasive mechanical ventilation due to impending respiratory failure, as evidenced by her point-of-care venous blood gas demonstrating acidosis with severe hypercapnia (pH of 7.29, pCO_2_ of 88 mmHg) and worsening mental status. Upon intubation, aeration in both the lungs significantly improved.

She was extubated to room air the following day after her blood gases normalized. She tolerated being extubated for several hours, but she soon showed signs of respiratory failure requiring BiPAP. Although initial presentation did not reveal any ptosis, physical exam after extubating demonstrated bilateral ptosis (half lidded). A complete neurological exam at that time revealed double vision, diminished strength throughout her extremities, and limited ability to raise her arms to a winged position which was not present at the time of admission. Upon further questioning, the family reported that for several months prior to admission, the patient also had vague concerns for fatigue, difficulty swallowing, and trouble keeping her eyelids fully open. Of note, the family did not consult their pediatrician about these fluctuating symptoms. The physical exam upon extubating and additional history provided by the parents prompted the team to pursue neurological causes for respiratory failure. Electromyography demonstrated normal limited nerve conduction, which ruled out demyelination disorders. Repetitive right axillary nerve stimulation demonstrated 30.1% change in amplitude from baseline to after exercise, consistent with myasthenia gravis. In addition, concentric electromyography on right deltoid, extensor digitorum communis, and trapezius muscles showed active denervation, suggesting a chronic process consistent with severe cases of neuromuscular junction disorders.

Working with a diagnosis of MG, she underwent a computerized tomographic scan which was negative for thymoma. She was found to be muscle-specific tyrosine kinase (MuSK) antibodies-positive, but negative for AChR antibodies and other autoimmune conditions. She was started on pyridostigmine (60 mg four times a day) and a five-day course of intravenous immunoglobulin (IVIG), both of which improved her symptoms within 24 hours. Throughout her hospital stay, the patient was on steroids, initially for presumed asthma exacerbation, and then was changed to a MG treatment dosage. She received a total of 1.5 mg/kg of steroids during her hospital stay. Her hospital stay was complicated by a prolonged need for respiratory support secondary to respiratory muscle weakness. She was discharged home with BiPAP for nighttime respiratory support and continued pyridostigmine and steroids. Per chart review, she is currently receiving IVIG every 4 weeks with outpatient physical therapy and close neurological follow-up.

She had one hospitalization for worsening respiratory distress approximately 3 weeks after initial discharge where the doses of pyridostigmine and steroids were increased. Her follow-up course was complicated by poor medication compliance issues due to side effects of steroids and pyridostigmine which require frequent dosage adjustments.

## 3. Discussion

Juvenile myasthenia gravis (JMG) is an autoimmune disorder whose pathophysiology has been well studied. Acetylcholine (ACh) is released into the synaptic cleft and travels to the acetylcholine receptor (AChR) sites causing depolarization. Autoantibodies against AChR, muscle-specific kinase (MuSK), and lipoprotein receptor-related membrane protein 4 (LRP-4), lead to decreased AChR activity through complement-mediated lysis of the postsynaptic membrane, increased degradation of the receptor, and direct inhibition of activity [3, 4]. MG positive for AChR antibodies is the most common, approximately 80% of all cases. MG positive for MuSK antibodies, as reported in this case, is less common, only 4% of all cases. The literature does not report significant difference among all the three subtypes in all the parameters studied including long-term prognosis and quality of life. MuSK antibodies are mainly IgG4, unlike the IgG1 and IgG3 anti-AChR antibodies, and are not complement-activating [5]. Furthermore, MG with MuSK antibodies is seen predominantly in females and commonly has atypical clinical features such as the selective facial, bulbar, neck, and respiratory muscle weakness and marked muscle atrophy, occasionally with relative sparing of ocular muscles [6, 7]. Respiratory crises are also more common. Weakness can involve muscles that are not usually symptomatic in MG such as paraspinal and upper esophageal muscles [8]. Anticholinesterase agents are less effective and induce frequent side effects [9]. Thymus histology is usually normal. [9] MG with MuSK antibodies has lower response with immunosuppressive treatment, and rituximab has a favorable response.

This case highlights the pattern of missed or delayed diagnosis of MG, so providers are aware of the various symptoms which patients present and how they were managed prior to diagnosing myasthenia. In other cases of unknown diagnosis of MG, children and adolescents presented to ED with unique symptoms such as a 4-year-old female with an 11-day history of eyelid swelling, redness, and drooping [10] and a 16-year-old African-American female with a history of chronic fatigue, depression, dysphagia, and tempomandibular joint dysfunction [11]. We believe that our patient had generalized MG as she had presented with acute respiratory failure requiring intubation in the first few hours of presentation, but after gathering further history, her presentation suggests she had ocular type for months prior to the presentation which progressed to generalized type with myasthenic crisis. This further demonstrates the need for a complete review of systems at all patient encounters and to continually reassess possible differentials if the patient is not responding in the expected manner to standard of care treatments.

Myasthenic crisis can be triggered by infections, physical or emotional stress, sleep deprivation, perimenstrual state, and various drugs. Clinicians should avoid drugs that affect neuromuscular transmission in the event of myasthenic crisis as they will exacerbate symptoms. Some of these drugs include macrolides, fluoroquinolones, voriconazole, high-dose beta blockers, and magnesium [7]. Magnesium induces a neuromuscular blockade at the presynaptic and postsynaptic endplate which inhibits the presynaptic release of ACh and desensitizes the postsynaptic membrane. Our patient received multiple doses of magnesium and corticosteroids during the initial management of asthma which could have triggered her myasthenic crisis resulting in complete respiratory failure. There have been several studies demonstrating a prolonged hospital course and severe respiratory failure requiring intubation all secondary to magnesium [8, 9, 12].

Once MG is suspected, clinicians can perform easy physical exam testing at the patient's bedside such as fatigability on sustained upgaze. A positive Cogan lid twitch and positive ice pack test may also demonstrate ocular symptoms. The “gold standard” for testing is an electrophysiological test called a single-fiber electromyography, which is performed on the orbicularis oculi. However, this is difficult to perform on children due to length and discomfort of testing [2]. Another method of electrophysiological testing calls for repetitive nerve stimulation with a positive result demonstrating a decremental response of 10% between the first and the fourth response which was positive in our patient [2]. Part of the work-up for MG also includes evaluation for thymoma, although rare in children [13].

Acetylcholinesterase inhibitors increase the amount of acetylcholine available to assist with muscle movements and can be used for testing and treatment of MG. Examples include edrophonium (tensilon) testing, neostigmine for young children, and pyridostigmine which is not performed in our patient [14]. As previously discussed, a patient must undergo evaluation for a thymoma. If found, a thymectomy may be required; however, the indications are less clear in the pediatric population [15]. In adolescents who have undergone a thymectomy, higher rates of remission have been reported [15]. In comparison, prepubertal children typically demonstrate a higher rate of spontaneous remission, which brings up concerns of premature immunosenescence with a thymectomy, although no studies have reported significant side effects [16].

Immunomodulators such as intravenous immunoglobulin or steroids may be helpful to reduce the circulating autoantibodies by decreasing B-cell antibody production and T-cell function [17]. They have better efficacy for short-term treatments and exacerbations than for long-term treatment [17]. Another way to reduce the circulating autoantibodies is through plasma exchange. This can directly filter these antibodies as well as cytokines from the patient's serum. It is very effective for improving strength within days during acute settings in prepubertal children. Plasma exchange and IVIG have proven similarly effective; however, use of plasma exchange is limited by availability, the need for large-bore venous access, and complications (hypotension, sepsis, pneumothorax, and pulmonary embolism) [17].

In summary, we share a case of a patient with a history of asthma that presented for acute onset dyspnea. The patient was presumptively managed for an asthma exacerbation with the standard asthma treatment regimen triggering a new onset myasthenic crisis. Since shortness of breath is a common chief complaint in the pediatric population with numerous differentials, it was not until the family shared additional history such as lid lag and chronic fatigue and clinical findings concerning for neuromuscular disease that appeared late that MG was considered as a possible diagnosis. A higher index of suspicion for MG and earlier divulgement of supportive history may help to make an earlier diagnosis, associated with better prognosis. This case acts as a reminder to all general pediatricians, emergency room providers, and critical care physicians to include neuromuscular causes for dyspnea and to exclude MG with good history and physical exam in all cases of status asthmaticus not responding to the standard regimen.

## Data Availability

Data sharing is not applicable to this article as no new data were created or analyzed in this study.
